# Genomic characteristics of ST6115 carbapenem-resistant *Klebsiella pneumoniae* coharboring *bla*_NDM-1_ and *bla*_IMP-4_

**DOI:** 10.3389/fmicb.2025.1545161

**Published:** 2025-02-05

**Authors:** Xiaofang Xie, Yaxuan Zhai, Zhichen Zhu, Feinan Qian, Jie Zhu, Qizhao Gao, Hong Du

**Affiliations:** ^1^Department of Clinical Laboratory, The Second Affiliated Hospital of Soochow University, Suzhou, China; ^2^MOE Key Laboratory of Geriatric Diseases and Immunology, The Second Affiliated Hospital of Soochow University, Suzhou, China; ^3^Department of Clinical Laboratory, Zhenjiang First People’s Hospital, Zhenjiang, China

**Keywords:** carbapenem-resistant *Klebsiella pneumoniae*, ST6115, IncHI5 plasmid, *bla*_NDM-1_, *bla*_IMP-4_

## Abstract

**Objectives:**

The aim of this study is to characterize the genomic features of ST6115 carbapenem-resistant *Klebsiella pneumoniae* (CRKP) co-harboring *bla*_NDM-1_ and *bla*_IMP-4_.

**Methods:**

The genome sequences of one ST6115 *Klebsiella pneumoniae* (KP) strain and 12 ST17 KP strain were obtained through whole genome sequencing (WGS). Subsequently, a phylogenetic analysis was employed to ascertain the clonal relationship of these strains. The antimicrobial susceptibility testing (AST) was evaluated through the application of the minimum inhibitory concentration (MIC) methodology by the broth microdilution method. Annotation and analysis of the genome enabled the identification of the plasmid structure and the comparative analysis of the genetic background. Finally, the conjugation transfer experiment was conducted to assess the transferability of the plasmid pHD8428-NDM-IMP.

**Results:**

A comparable phylogenetic analysis revealed that ST6115 HD8428 and the majority of ST17 strains (9/12) were clustered on the same clade, which suggests a close relationship between two ST types. Additionally, HD8428 showed particularly close genetic similarity to HD11490, with a single-nucleotide polymorphism (SNP) difference of only 273. The analysis of the antibiotic resistance genes carried by the 13 strains revealed that all strains carry extended-spectrum β-lactamase (ESBL) genes. AST revealed that HD8428 exhibited resistance to a diverse range of antibiotics. The *bla*_NDM-1_ and *bla*_IMP-4_ genes were identified as being located on the plasmid pHD8428-NDM-IMP. Further analysis demonstrated that the *bla*_NDM-1_ gene was present on ΔTn*125*, while the *bla*_IMP-4_ gene was located on In1377-2. The results of the conjugation experiment indicated that plasmid pHD8428-NDM-IMP may pose a risk for the transmission of antibiotic resistance in healthcare settings.

**Conclusion:**

We report a clinical ST6115 strain HD8428 and identify the coexistence of *bla*_NDM-1_ and *bla*_IMP-4_ in the IncHI5 type plasmid pHD8428-NDM-IMP. HD8428 was resistant to a wide range of antibiotics and harbored the transmissible plasmid pHD8428-NDM-IMP, which made it a potential threat to public health. Our study indicates that the healthcare system and services should remain vigilant regarding the spread and prevalence of ST6115.

## Introduction

*Klebsiella pneumoniae* (KP) is a causative agent associated with a range of infections, including pneumonia, sepsis, urinary tract infection, bacteremia and so on (Paczosa and Mecsas, 2016). The extensive utilization of antibiotics has unquestionably contributed to the enhanced resistance of KP to multiple antibiotics, which presents a significant challenge to the effective treatment of pathogens and has garnered considerable attention. The World Health Organization (WHO) has identified multidrug-resistant (MDR) Gram-negative bacteria (GNB) as a pathogen in urgent need of new antibiotics (Tacconelli et al., 2018).

The majority of MDR-KP isolates produce extended-spectrum β-lactamases (ESBLs) and/or carbapenemases in collaboration with other antibiotic resistance mechanisms (Woodford et al., 2011; Bialek-Davenet et al., 2014). The emergence of ESBLs-producing KP is a global phenomenon, with high mortality rates occurring in the infection (Shu et al., 2019). Carbapenem-resistant *Klebsiella pneumoniae* (CRKP) is a consequence of excessive use of carbapenems in the treatment of ESBL-producing KP (Arato et al., 2021). The emergence of CRKP has become a significant concern for both patients and clinicians. In particular, the presence of CRKP has been linked to an approximately 40% increase in mortality rates in hospitals (Zhou et al., 2020).

The clinically important MDR-KP isolates harbor plasmid-borne genes encoding a wide variety of carbapenemases, including class A (KP carbapenemase, KPC), class D (oxacillinase, OXA), and class B, such as New Delhi metallo-β-lactamase (NDM), imipenemase (IMP), and Verona integron-encoded metallo-β-lactamase (VIM) (Jean et al., 2022). KPC is the most common in China, followed by NDM and IMP (Wang et al., 2024). Currently, the increasing prevalence of carbapenem-resistant *Enterobacteriaceae* (CRE) strains in China is primarily attributable to the pervasive transmission of conservative mobile elements harboring the *bla*_NDM_ gene (Zhang et al., 2017). Moreover, the literature indicated that the prevalence of IMP-4 in China has reached a critical level that necessitates the expansion of surveillance to prevent the further spread of CRKP (Zheng et al., 2023). Furthermore, the co-carrying of more kinds of resistance genes has the potential to facilitate the dissemination of these genes in MDR bacteria, thereby complicating the management of resistant genes (Zhang et al., 2021).

The ST6115 strain was identified in 2022. There is a paucity of reports of the ST6115 KP, which is analogous to ST17 and differs from it by a single allele difference in *tonB* gene. ST17 is strongly associated with a MDR phenotype, which contributes to challenging hospital-acquired infections (Hetland et al., 2023). Therefore, a comparison with ST17 allows for an assessment of the potential spread of ST6115.

In the course of our investigation, we identified a strain of KP belonging to ST6115 and co-harbored *bla*_NDM-1_ and *bla*_IMP-4_. This strain may serve as a potential model for deep analysis of the transmission pathway and genetic context. Therefore, our findings provide valuable insights into the carbapenemases genes transfer and offer a foundation for the development of prevention and control strategies.

## Materials and methods

### Strains collection and antimicrobial susceptibility testing

Thirteen KP strains were collected from six tertiary health care hospitals from different cities in China between 2017 and 2022. These cities are located in North China (Beijing), East China (Suzhou), South China (Guangzhou), Southwest China (Chengdu, and Kunming), and Northwest China (Yinchuan), respectively. We retrospectively collected all CRKP strains and the clinical data from these hospitals. Bacterial species identification was performed using matrix-assisted laser desorption time of flight mass spectrometry (MALDI-TOF-MS). Subsequently, genomic DNA was extracted from the bacteria to facilitate further determination of the ST type and the resistant genes harbored by these strains. The bacterial strain collection was described in our previous study (Zhu et al., 2023). We performed antimicrobial susceptibility testing (AST) using the standard broth microdilution technique following the CLSI guidelines (Humphries et al., 2021).

### Whole-genome sequencing

Genomic DNA from KP strains was extracted using the Omega Bio-Tek Bacterial DNA Kit (Doraville, GA, United States). Whole-genome sequencing (WGS) of the 13 isolates was carried out on the Illumina NovaSeq 6000 platform (Illumina Inc., San Diego, CA, United States) using a 350 bp paired-end protocol. The filtered reads were assembled *de novo* into contigs with *SPAdes* 3.11. The complete genome of isolate HD8428 was sequenced using a sheared DNA library with an average fragment size of 10 kb on a Nanopore PromethION sequencer (Oxford Nanopore Technologies, United Kingdom). And paired-end short reads from Illumina sequencing were employed to correct the longer Nanopore reads. The corrected Nanopore reads were then assembled de novo using *Unicycler* v0.4.9. The methodology is based on our previous study (Wen et al., 2022).

### Genome analysis

Open reading frames (ORFs) and pseudogenes were identified through *RAST 2.0*, with additional confirmation via *BLASTp/BLASTn* searches. Multilocus sequence typing (MLST), along with the annotation of resistance genes, mobile elements, and other relevant features, was conducted using various online databases, including *PubMLST*, *CARD*, *ResFinder*, *PlasmidFinder*, *ISfinder*, and *INTEGRALL*. Sequence comparisons, both pairwise and multiple, were carried out using *BLASTn*. Gene organization diagrams were generated using *Danmel* scripts and visualized with *Inkscape 1.0.1*. The Sankey diagram was created using SankeyMATIC. The details were described in our previous study (Fu et al., 2024).

### Phylogenetic analysis

A phylogenetic analysis was performed on 13 KP strains. The core single nucleotide polymorphisms (SNPs) were identified by *Mummer 3.25*. A maximum-likelihood phylogenetic tree was constructed using *MEGAX 10.1.8* based on the core SNPs with a bootstrap iteration of 1,000, and displayed using iTOL. The details were outlined in our earlier study (Fu et al., 2024).

### Conjugal transfer

We performed conjugation experiments between the HD8428 donor strain and the *E. coli* J53 recipient strain (resistant to NaN_3_), using same volume mixed. Then they were incubated for 24 h at 24°C. Transconjugants were selected on agar plates containing 2 μg/mL meropenem and 200 μg/mL NaN3. PCR analysis was then conducted to confirm the presence of the conjugants, following the previously described protocol (Fu et al., 2024). The primer sequences we used are as follows: NDM-1 (F: GAATGGCTCATCACGATCATGC, R: CGGTTTGATCGTCAGGGATGG), IMP-4 (F: GAAGGCGTTTATGTTCATACTTCGT, R: CTTGGAACAACCAGTTTTGCCT).

### Growth assay

Strains were cultured overnight in 3 mL of LB with or without meropenem, shaking at 200 rpm and 37°C, then diluted to an OD_600_ of 0.3. And 2 μL of that liquid was added to 200 μL of LB in a 96-well plate, with triplicate wells for each condition. The culture density was monitored every 30 min for 16 h by measuring OD_600_ with shaking at 200 rpm and 37°C using a FLUOstar Omega reader (BMG Labtech, Germany). Growth curves were generated and analyzed using *GraphPad Prism 5.0* (GraphPad Software, Inc.), with statistical significance determined by two-way ANOVA (*p* < 0.05).

### Nucleotide sequence accession numbers

The 12 draft genome sequences of ST17 strains and the complete genome sequence of ST6115 strain were, respectively, submitted to GenBank under BioProject PRJNA1189667 and PRJNA1189857.

## Results

### The clinical information and serotypes of 13 strains in this study

**Figure 1:**
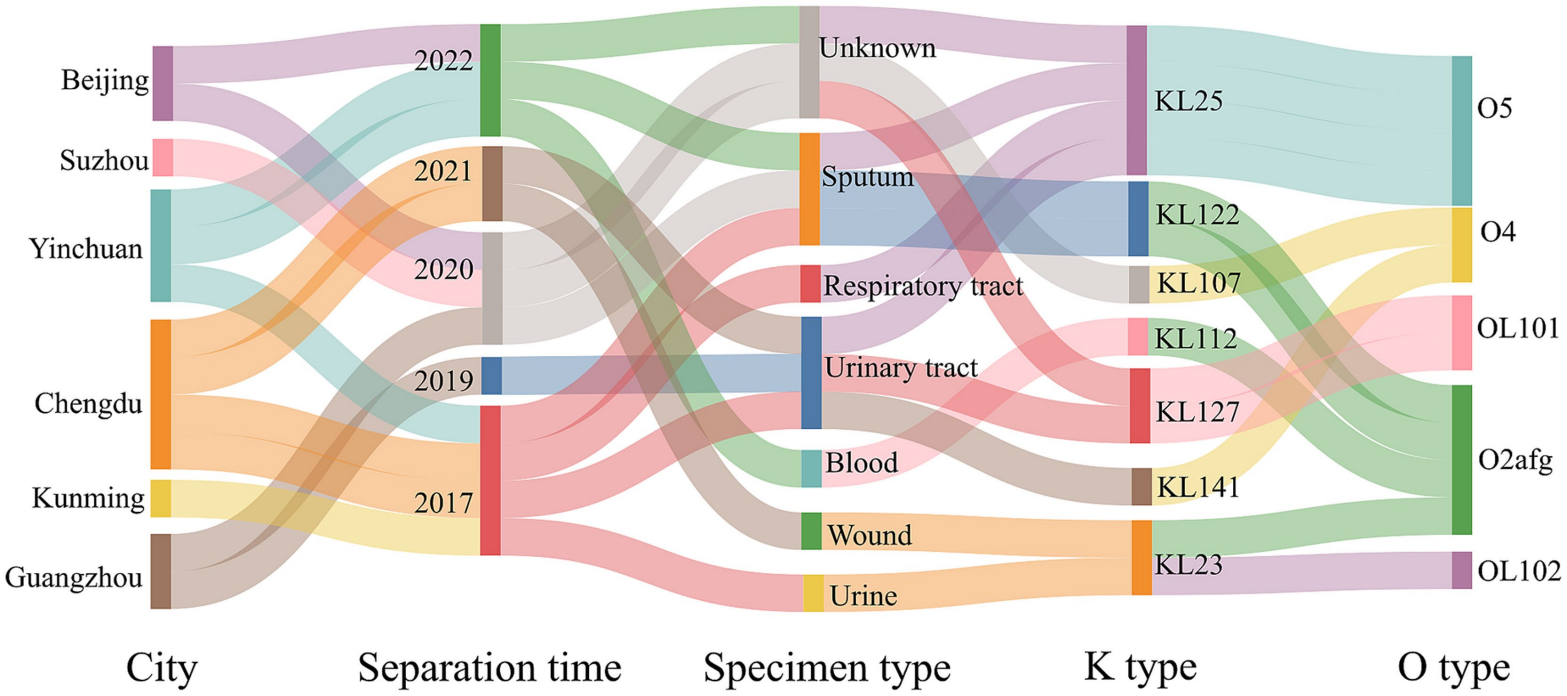
The clinical information and serotypes of ST6115 and 12 ST17 isolates. Sankey diagram showing 13 strains from different cities, separated by time and specimen types in China (2017–2022), with information on capsular polysaccharide and lipopolysaccharide O types.

A total of 12 ST17 KP isolates were collected in the domestic multicenter surveillance study. During the course of our collection, we fortuitously encountered a ST6115 strain designated HD8428. The clinical information and serotypes of the 13 strains were initially organized and presented in a Sankey diagram (Figure 1). These isolates were collected from six cities over the past 5 years and were isolated from sputum, urinary tract and other clinical specimens. Based on capsular serotypes, seven distinct types were identified, with KL25 (4/13) being the most prevalent. Additionally, a total of five types were classified based on lipopolysaccharide O antigens, with O5 (4/13) and O2afg (4/13) being the most prevalent. It was notable that all four KL25 strains exhibit an O5 serotype, while all two KL127 strains are of OL101 serotype.

### Comparative analysis of phylogenetic trees between ST6115 and ST17

**Figure 2:**
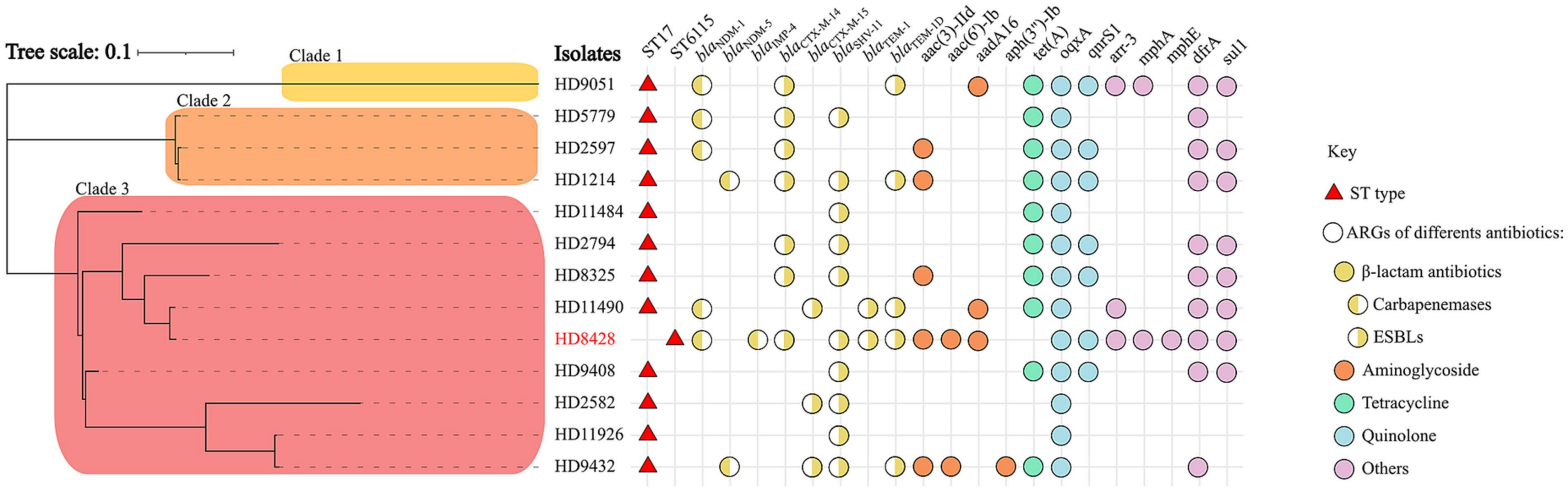
A maximum-likelihood phylogenetic tree of ST6115 HD8428 and 12 ST17 isolates. The red triangles represent ST types, and the circles represent ARGs of different kinds of antibiotics: yellow for β-lactam antibiotics, orange for aminoglycoside, green for tetracycline, blue for quinolone, and purple for other antibiotics. Among these, the resistance genes for β-lactam antibiotics are categorized into two types: the left semicircle represents carbapenemases genes, while the right semicircle represents ESBLs genes. Bar corresponds to scale of sequence divergence.

Given the paucity of reports on this particular class of ST6115, we sought to undertake a comparative analysis between ST6115 and ST17, with a view to elucidating the distinctive characteristics of them. To compare the clonality of two types of KP in China, a phylogenetic analysis based on core SNPs was performed on Figure 2. The phylogenetic tree revealed that 13 KP were divided into three major separately clustering clades. The HD8428 and the other nine KP strains were found to co-exist on clade 3. Of these, HD11490 exhibited the lowest degree of divergence to HD8428 (SNP = 273). The findings suggest that ST6115 exhibits a high degree of similarity with ST17. Additionally, HD9051 was distinctly situated on clade 1, while the remaining three strains exhibiting close relationship were located on clade 2. However, the core SNPs of 13 strains were further pairwise compared, with the smallest core SNP difference being 119, suggesting that these strains did not belong to the same clone.

### Antimicrobial genetic characteristics of 13 strains

In this study, the presence of antibiotic resistance genes was analyzed in 13 strains. Among them, most strains (8/13) carried carbapenemase-encoding genes, including five *bla*_NDM-1_, two *bla*_NDM-5_, and one *bla*_IMP-4_. Of note, HD8428 co-carried *bla*_NDM-1_ and *bla*_IMP-4_ (Figure 2). The findings indicated that strains carrying the *bla*_NDM-1_ gene constitute approximately half of the ST17 group.

Furthermore, additional antimicrobial resistance genes (ARGs) were identified. The antimicrobial genetic analysis indicated that all isolates harbored different kinds of extended-spectrum beta-lactamases (ESBLs), including *bla*_CTX-M-14_, *bla*_CTX-M-15_, *bla*_TEM-1_ *bla*_TEM-1D_ and *bla*_SHV-11_ (Figure 2). Of the 13 strains, *bla*_SHV-11_ and *bla*_CTX-M-14_ had the highest prevalence of 76.9 and 53.8%, respectively. The isolates also harbored for other resistance genes such as *aac(3)-IId*, *aac(6′)-Ib*, *aadA16* and *aph(3″)-Ib* for aminoglycoside, *tet (A)* for tetracycline, *aac(6′)-Ib-cr*, *oqxA* and *qnrS1* for fluoroquinolones and so on. The *oqxA* gene was identified in all strains.

### Antimicrobial resistance profiles

AST is used to detect bacterial resistance to antibiotics *in vitro*. The AST results of ST6115 HD8428 and the 12 ST17 strains were shown in Table 1. HD8428 was resistant to carbapenems, like meropenem. It was also resistant to cephalosporin, like ceftazidime. And HD8428 was resistant to aminoglycoside such as gentamycin and amikacin. HD8428 retained resistance to fluoroquinolones, like ciprofloxacin. But HD8428 was susceptible to tetracycline, like tigecycline. Our results suggest that HD8428 is MDR.

**Table 1:** Susceptibilities of 15 strains to six antimicrobials.

Isolates	Antimicrobial MIC (mg/L)					
	MEM^a^	CAZ	GEN	AMK	TGC	CIP
HD9051	**16** ^b^	**≥256**	1	8	2	**≥256**
HD5779	**16**	**≥256**	1	2	1	≤0.5
HD2597	**16**	**≥256**	**≥256**	4	≤0.5	≤0.5
HD1214	**32**	**≥256**	**128**	2	2	≤0.5
HD11484	≤0.5	≤0.5	2	4	2	≤0.5
HD2794	≤0.5	**16**	≤0.5	1	2	**1**
HD8325	1	8	**256**	2	**16**	**4**
HD11490	**16**	**≥256**	1	4	1	≤0.5
HD8428	**64**	**≥256**	**256**	**16**	≤0.5	**1**
HD9408	≤0.5	**128**	1	4	**16**	**4**
HD2582	≤0.5	≤0.5	1	4	≤0.5	≤0.5
HD11926	≤0.5	≤0.5	1	4	≤0.5	≤0.5
HD9432	**64**	**≥256**	**≥256**	4	2	**16**
J53	≤0.5	≤0.5	1	4	≤0.5	≤0.5
J53 + pDH8428-NDM-IMP	**32**	**≥256**	**128**	**16**	≤0.5	≤0.5

a MEM, meropenem; CAZ, ceftazidime; GEN, gentamycin; AMK, amikacin; TGC, tigecycline; CIP, ciprofloxacin.

b The bold numbers were interpreted as resistant (R).

In general, the AST of all the strains can be largely attributed to the resistant genes they harbor. The presence of a specific carbapenemase gene rendered seven strains resistant to both carbapenems and ceftazidime. Conversely, when only ESBLs were present, HD2794 and HD9408 exhibited resistance to ceftazidime. Furthermore, all strains that carried aminoglycoside resistance genes were resistant to gentamycin and amikacin. And the *tet (A)* gene has been identified as a resistance factor for tigecycline in both the HD8325 and HD9408 isolates. Furthermore, our findings indicate that the presence of resistance genes in bacterium does not necessarily exhibit resistance to the corresponding antibiotic. This phenomenon is particularly evident in strains carrying the *oqxA* and *qnrS1* genes. Some strains that carried quinolone resistance genes but remained sensitive to quinolone antibiotics. This could be due to low or lack of expression of the resistance genes.

### Characterization of plasmid pHD8428-NDM-IMP, *bla*_NDM-1_ region and *bla*_IMP-4_ region

**Figure 3:**
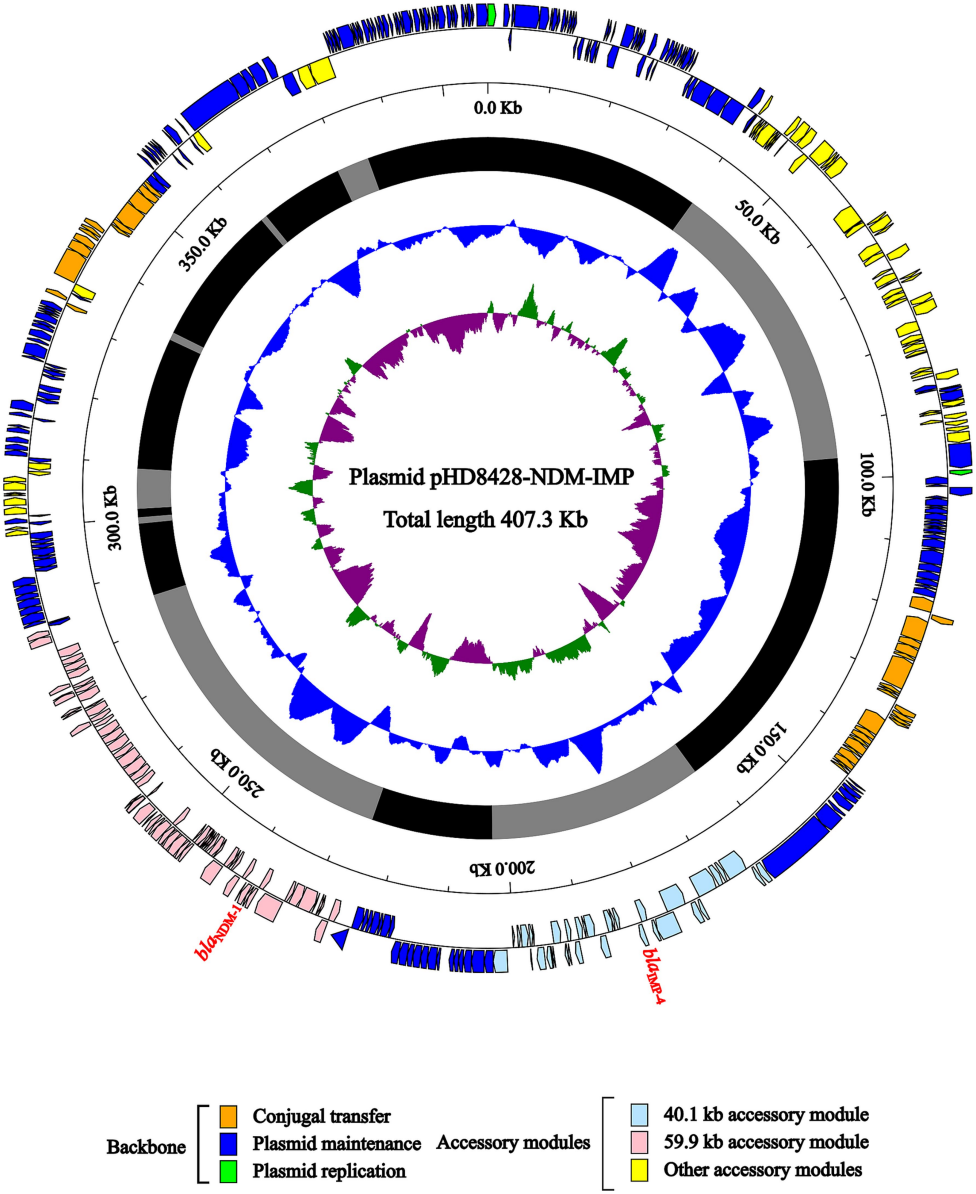
Schematic diagram of IncHI5-type plasmid pHD8428-NDM-IMP. The presentation of genes of different functions is indicated by arrows and is distinguished by a variety of colors. The circles indicate the predicted coding sequences, with the scale in 10 kb shown outside, the backbone (black) and accessory module (gray) regions shown next, GC content and GC skew [(G − C)/(G + C)] shown inside.

In HD8428, the *bla*_NDM-1_ and *bla*_IMP-4_ genes were both carried by plasmid pHD8428-NDM-IMP, which was identified as an IncHI5 plasmid (Figure 3). The pA324-IMP plasmid was employed as a reference plasmid (Liang et al., 2018), belonging to the IncHI5 plasmid type and bearing the accession number MF344566. The reference plasmid encodes two replication proteins, designated RepFIB and RepHI5B. In order to annotate the unknown pHD8428-NDM-IMP plasmid, a BLAST comparison was performed with the well-characterized pA324-IMP plasmid. The results revealed that two replication proteins present in the pHD8428-NDM-IMP plasmid, which were consistent with the reference plasmid. Furthermore, a comparison of the backbone regions of the pHD8428-NDM-IMP plasmid and the reference plasmid revealed a high degree of similarity, with 95% coverage and 99.5% nucleotide identity. Based on these results, the pHD8428-NDM-IMP plasmid is identified as an IncHI5 plasmid. The backbone region of plasmid pHD8428-NDM-IMP was dispersed, which was caused by the insertion of multiple exogenous DNAs at different sites. In essence, the backbone region is roughly separated by 2 principal accessory module (a 59.9 kb accessory module carrying the *bla*_NDM-1_ gene and a 40.1 kb accessory module carrying the *bla*_IMP-4_ gene), and other six scattered accessory modules (Figure 3).

**Figure 4:**
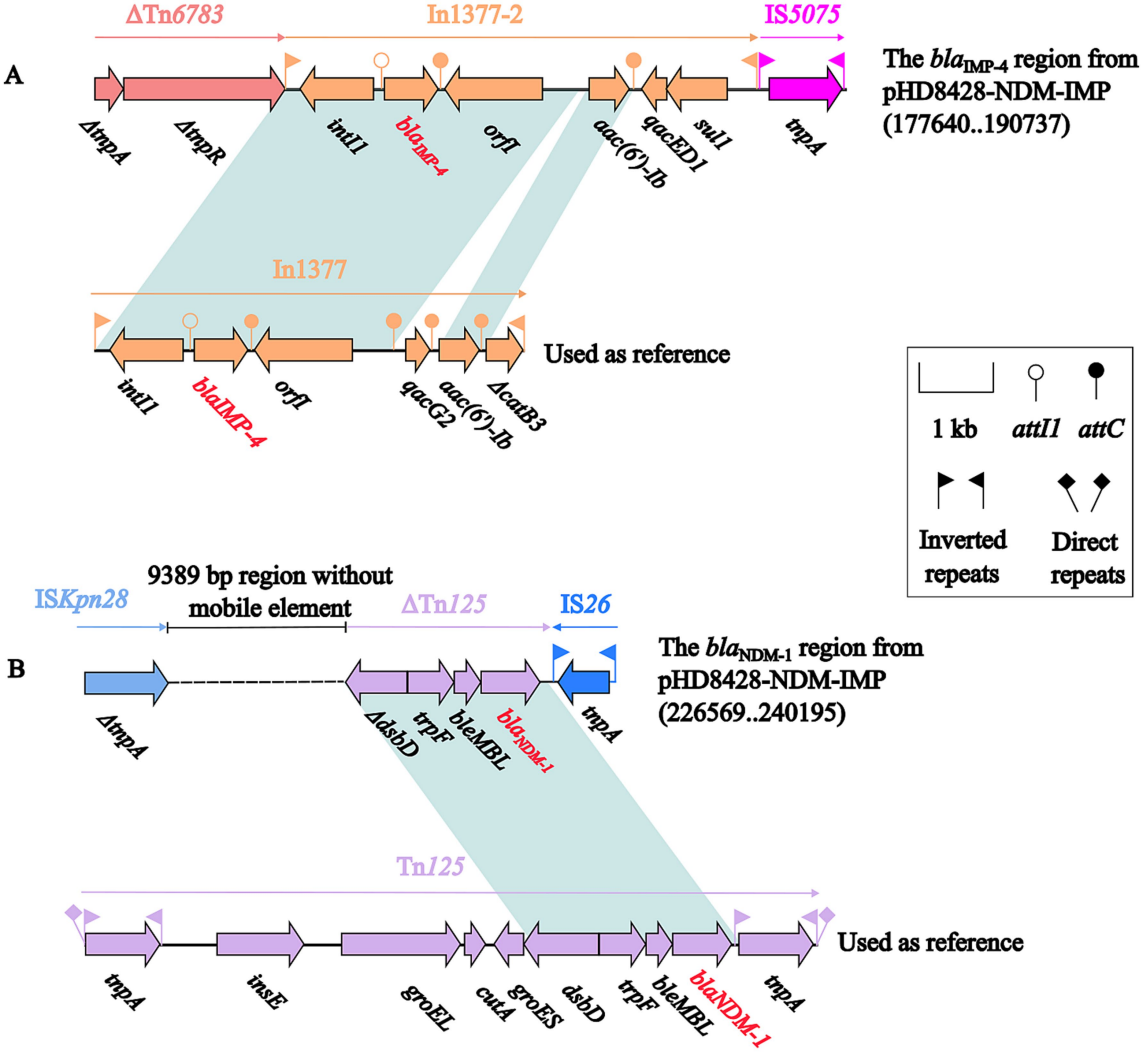
The comparative analysis of the genetic background of the IMP-4 region and In1377 **(A)**, NDM-1 region and Tn125 **(B)**. The representation of genes is indicated by arrows. Mobile genetic elements are categorized according to their functional classification and presented in a color-coded format. The dashed line in the figure represents a shortened version of the actual length. Shading denotes regions of homology (light blue: ≥99% nucleotide identity). The accession numbers of In1377 and Tn125 used as reference are MF344567 and JN872328, respectively.

Further, a comparative analysis of the environment surrounding the *bla*_NDM-1_ and *bla*_IMP-4_ gene were, respectively, carried out. A 10.3 kb IMP-4 region was detected in the 40.1 kb accessory module (Figure 4A). The *bla*_IMP-4_ genes were carried by the In1377-2. Compared to In1377, the gene cassette of In1377-2 lacks the *qacG2* and Δ*catB3* genes, while the coverage and nucleotide identity of rest sequences were 100%. And In1377-2 was surrounded by two genetic elements, ΔTn*6738* and IS*5075*. The other 13.6 kb NDM-1 region was also identified in the 59.9 kb accessory module (Figure 4B). On this region, *bla*_NDM-1_ gene was carried by ΔTn*125*. In comparison with the complete Tn*125*, ΔTn*125* is 100% identical to it, but contains only Δ*dsbD*, *trpF*, *ble*_MBL_ and *bla*_NDM-1_ genes.

### The conjugation capacity of the pHD8428-NDM-IMP plasmid

**Figure 5:**
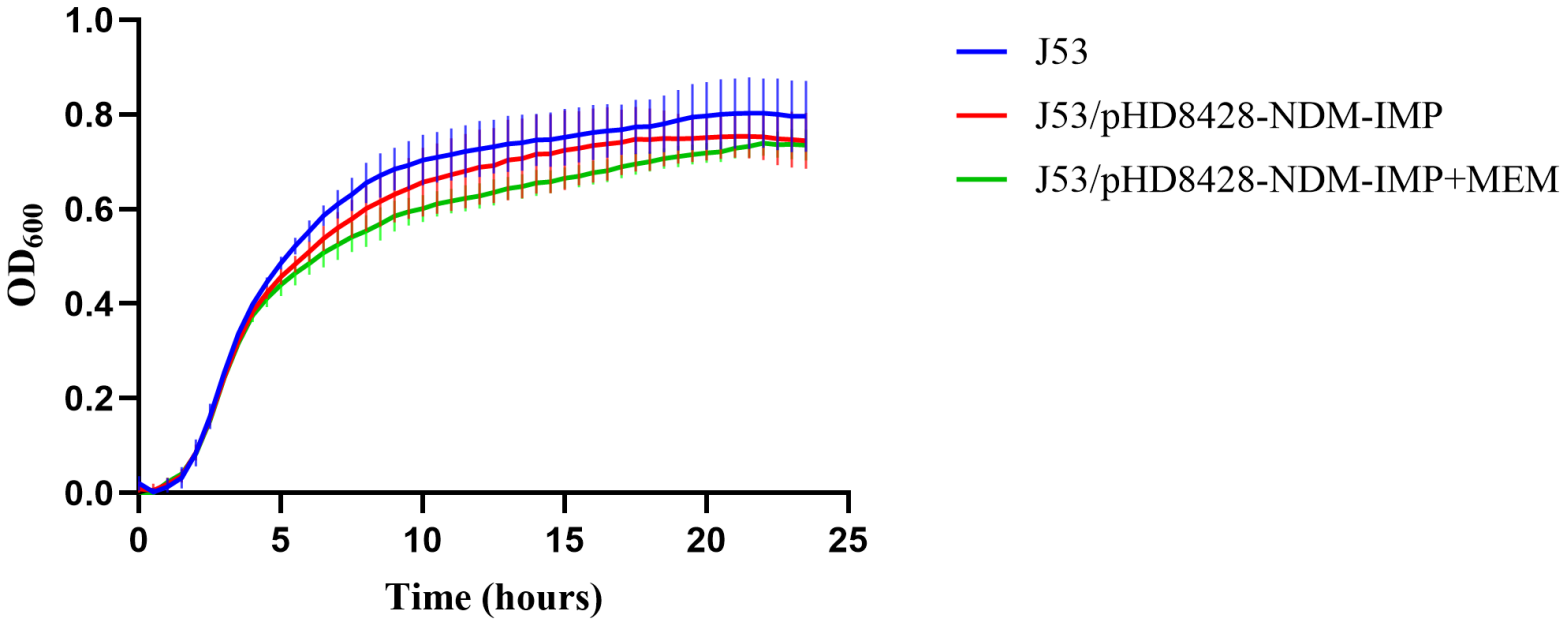
Growth curve comparison. Growth curves of recipient strain *E. coli* J53 and the transconjugants harboring *bla*_NDM-1_ and *bla*_IMP-4_ co-carried IncHI5-type plasmid pHD8428-NDM-IMP with and without meropenem (value of *p* > 0.05). All data are presented as mean ± SD (*n* = 3). J53 means the recipient strain *E. coli* J53. J53/pHD8428-NDM-IMP means the transconjugant harboring IncHI5-type plasmid pHD8428-NDM-IMP in antibiotic-free LB broth. J53/pHD8428-NDM-IMP+MEM means the transconjugant harboring IncHI5-type plasmid pHD8428-NDM-IMP in LB containing meropenem.

The IncHI5-type plasmid pHD8428-NDM-IMP could be transferred into *E. coli* J53 from strain HD8428 through conjugal transfer experiment. In order to evaluate the influence of the IncHI5-type plasmid on the growth of the strain, a comparison was made between the growth of *E. coli* J53 and *E. coli* J53 transconjugants of the IncHI5-type plasmid pHD8428-NDM-IMP (Figure 5). The difference between the growth curves of J53 and J53/pHD8428-NDM-IMP was not statistically significant (*p* > 0.05) in all conditions, including those with and without meropenem restriction. The results indicate that the acquisition of IncHI5-type plasmid pHD8428-NDM-IMP had minimal impact on the growth of the parental strain.

Furthermore, a comparative analysis of the AST was conducted between the J53 and J53/pHD8428-NDM-IMP strains (Table 1). It was observed that the recipient strain, through the acquisition of the plasmid, exhibited an increase in resistance to carbapenems, cephalosporins, and aminoglycosides. This phenomenon can be attributed to the presence of the *bla*_NDM-1_, *bla*_IMP-4_ and *aac(6′)-Ib* genes on the pHD8428-NDM-IMP plasmid (Figure 4). This indicates that upon transfer of the plasmid, the recipient strain exhibits enhanced resistance to the several kinds of antibiotics.

## Discussion

In the present study, we report a rare clinical strain of KP that belongs to ST6115 and carries two carbapenemase genes. We analyze this pathogen from different perspectives and elucidate the potential dangers. ST6115 has been classified in 2022, and has not been reported in any study. To our knowledge, this report is the first study of clinical ST6115. Given the minimal housekeeping gene divergence between ST6115 and ST17, we performed comparable phylogenetic analysis between them. The results demonstrate a close relationship between the two kinds of ST. In previous studies, the potential harm of ST17 KP to the public was demonstrated primarily through its capacity to carry CTX-related plasmids. It has been documented that ST17 KP producing CTX-M-15 has emerged in Canada, Spain, Norway and other countries (Löhr et al., 2015). Furthermore, ST17 has been identified as a high-risk clone that is responsible for the emergence and dissemination of CTX-like genes (Elias et al., 2023). Our study also produced similar findings. In our domestic multicenter surveillance study, the prevalence of ESBL genes in ST17 strains was notably high (100%), especially for *bla*_CTX-M-14_ (6/12) and *bla*_SHV-11_ (9/12) (Figure 2). Moreover, both of these resistance genes were also detected in the ST6115 strain. Furthermore, ST6115 exhibits a high degree of similarity to ST17, suggesting a potential correlation between their emergences. Therefore, this result serves to alert the necessity of preventing the transmission of ST6115.

In addition, our results indicated that KL25 and O5 are co-occurring and have the highest prevalence among the ST17 strains, which is consistent with previous studies (Hetland et al., 2023). However, ST6115 HD8428 does not belong to KL25/O5. This suggests that ST6115 may have other disseminated serotypes.

The prevalence of MBL producers has increased considerably (Sader et al., 2023). The *bla*_NDM-1_ gene is highly prevalent in numerous countries, for examples an epidemic outbreak of NDM-1 KP in Portugal (Novais et al., 2022), South Africa (Magobo et al., 2023), Germany (Sandfort et al., 2022) and so on. However, it should be noted that NDM-1 is not the most prevalent subtype detected in China (Han et al., 2020). IMP-4 is markedly prevalent and exhibits the highest detection rate of IMP subtypes in China (Jing et al., 2022; Wang et al., 2018). The production and transfer of these two enzymes has resulted in an increased tolerance of KP to carbapenems. The production and transfer of *bla*_IMP-4_ and *bla*_NDM-1_ have both actively contributed to the accelerated emergence of carbapenem resistance in an increasing number of KP.

The *bla*_NDM-1_ has been documented in the context of a range of plasmids, including IncM2 (Lopez-Diaz et al., 2022), IncHI2 (Fu et al., 2023), IncX3 (Elshamy et al., 2023), and IncA/C2 (Kubota et al., 2023). Additionally, there is some literatures attesting to the presence of *bla*_NDM-1_ on the IncHI5 plasmid (Liu et al., 2021; Zhu et al., 2020). A number of studies have reported that IMP-4 is present on a range of plasmids, including the IncHI2 (Roberts et al., 2020), IncN (Liu et al., 2024), and IncHI5 plasmids (Zheng et al., 2023). Additionally, some studies have documented instances of *bla*_NDM-1_ and *bla*_IMP-4_ co-existence within the same bacterial strain. These instances have been observed on the same plasmid (Jia et al., 2022; Xiao et al., 2022; Wang et al., 2022), on different plasmids (Ma et al., 2024; Liu et al., 2018), or on segments of the plasmid integrated with the chromosome (Shi et al., 2024). Thus, the co-presence of *bla*_NDM-1_ and *bla*_IMP-4_ on the IncHI5 plasmid is not an unexpected occurrence. However, this phenomenon that *bla*_NDM-1_ and *bla*_IMP-4_ co-exist in IncHI5 plasmid has not been documented in previous existing literature.

Despite the comprehensive bioinformatics analysis and experimental research conducted on a strain belonging to ST6115 and carrying resistance genes, this study is still limited by the lack of a sufficiently large number of strains to determine the generalizability of our findings. Moving forward, we will continue to monitor this phenomenon with the aim of improving the accuracy of our conclusions by increasing the sample size, thereby providing more precise results for clinicians and researchers.

In our study, we report a clinical strain of ST6115 CRKP co-carrying *bla*_NDM-1_ and *bla*_IMP-4_. There is a well-founded apprehension that an epidemic of ST6115 may occur as a connection of the emergence and subsequent dissemination of ST17 in China. Therefore, controlling antimicrobial resistance and close monitoring is essential and critical. It is crucial to acknowledge that the plasmid bearing *bla*_NDM-1_ and *bla*_IMP-4_ has the capacity to transfer, which underscores the importance of infection control measures inspired by CRE in clinical settings.

## Data Availability

The datasets presented in this study can be found in online repositories. The names of the repository/repositories and accession number(s) can be found in the article/supplementary material.

## References

[ref1] Arato V. Raso M. M. Gasperini G. Berlanda Scorza F. Micoli F. (2021). Prophylaxis and treatment against *Klebsiella pneumoniae*: current insights on this emerging anti-microbial resistant global threat. Int. J. Mol. Sci. 22:4042. doi: 10.3390/ijms22084042, PMID: 33919847 PMC8070759

[ref2] Bialek-Davenet S. Criscuolo A. Ailloud F. Passet V. Jones L. Delannoy-Vieillard A. S.. (2014). Genomic definition of hypervirulent and multidrug-resistant *Klebsiella pneumoniae* clonal groups. Emerg. Infect. Dis. 20, 1812–1820. doi: 10.3201/eid2011.140206, PMID: 25341126 PMC4214299

[ref3] Elias R. Spadar A. Hendrickx A. P. A. Bonnin R. A. Dortet L. Pinto M.. (2023). Emergence of KPC-3- and OXA-181-producing ST13 and ST17 *Klebsiella pneumoniae* in Portugal: genomic insights on national and international dissemination. J. Antimicrob. Chemother. 78, 1300–1308. doi: 10.1093/jac/dkad093, PMID: 36999363

[ref4] Elshamy A. A. Saleh S. E. Aboshanab K. M. Aboulwafa M. M. Hassouna N. A. (2023). Transferable IncX3 plasmid harboring *bla*_NDM-1_, *ble*_MBL_, and *aph(3′)*-VI genes from *Klebsiella pneumoniae* conferring phenotypic carbapenem resistance in *E. coli*. Mol. Biol. Rep. 50, 4945–4953. doi: 10.1007/s11033-023-08401-9, PMID: 37081308 PMC10209314

[ref5] Fu S. Jin S. Ge H. Xu Z. Jiao X. Chen X. (2023). First detection of *bla*_NDM-1_-haboring IncHI2 plasmid in *Escherichia coli* strain isolated from goose in China. Foodborne Pathog. Dis. 20, 244–250. doi: 10.1089/fpd.2022.0071, PMID: 37335912

[ref6] Fu H. Zhu Z. Wang X. Lv J. Zhu J. Chen L.. (2024). Emergence of *bla*_NDM-1_-carrying *Enterobacter chengduensis* in China. Front. Microbiol. 15:1404996. doi: 10.3389/fmicb.2024.1404996, PMID: 39206374 PMC11350614

[ref7] Han R. Shi Q. Wu S. Yin D. Peng M. Dong D.. (2020). Dissemination of carbapenemases (KPC, NDM, OXA-48, IMP, and VIM) among carbapenem-resistant *Enterobacteriaceae* isolated from adult and children patients in China. Front. Cell. Infect. Microbiol. 10:314. doi: 10.3389/fcimb.2020.00314, PMID: 32719751 PMC7347961

[ref8] Hetland M. A. K. Hawkey J. Bernhoff E. Bakksjø R. J. Kaspersen H. Rettedal S. I.. (2023). Within-patient and global evolutionary dynamics of *Klebsiella pneumoniae* ST17. Microb. Genom. 9:mgen001005. doi: 10.1099/mgen.0.001005, PMID: 37200066 PMC10272876

[ref9] Humphries R. Bobenchik A. M. Hindler J. A. Schuetz A. N. (2021). Overview of changes to the clinical and laboratory standards institute performance standards for antimicrobial susceptibility testing, M100, 31st edition. J. Clin. Microbiol. 59:e0021321. doi: 10.1128/JCM.00213-21, PMID: 34550809 PMC8601225

[ref10] Jean S. S. Harnod D. Hsueh P. R. (2022). Global threat of carbapenem-resistant Gram-negative bacteria. Front. Cell. Infect. Microbiol. 12:823684. doi: 10.3389/fcimb.2022.823684, PMID: 35372099 PMC8965008

[ref11] Jia X. Jia P. Zhu Y. Yu W. Li X. Xi J.. (2022). Coexistence of *bla*_NDM-1_ and *bla*_IMP-4_ in one novel hybrid plasmid confers transferable carbapenem resistance in an ST20-K28 *Klebsiella pneumoniae*. Front. Microbiol. 13:891807. doi: 10.3389/fmicb.2022.891807, PMID: 35711757 PMC9194606

[ref12] Jing N. Yan W. Zhang Q. Yuan Y. Wei X. Zhao W.. (2022). Epidemiology and genotypic characteristics of carbapenem resistant Enterobacterales in Henan, China: a multicentre study. J. Glob. Antimicrob. Resist. 29, 68–73. doi: 10.1016/j.jgar.2022.01.029, PMID: 35134552

[ref13] Kubota H. Nakayama T. Ariyoshi T. Uehara S. Uchitani Y. Tsuchida S.. (2023). Emergence of *Phytobacter diazotrophicus* carrying an IncA/C(2) plasmid harboring *bla*_NDM-1_ in Tokyo, Japan. mSphere 8:e0014723. doi: 10.1128/msphere.00147-23, PMID: 37449846 PMC10449528

[ref14] Liang Q. Jiang X. Hu L. Yin Z. Gao B. Zhao Y.. (2018). Sequencing and genomic diversity analysis of IncHI5 plasmids. Front. Microbiol. 9:3318. doi: 10.3389/fmicb.2018.03318 30692976 PMC6339943

[ref15] Liu Z. Chen R. Xu P. Wang Z. Li R. (2021). Characterization of a *bla*_NDM-1_-bearing IncHI5-like plasmid from *Klebsiella pneumoniae* of infant origin. Front. Cell. Infect. Microbiol. 11:738053. doi: 10.3389/fcimb.2021.738053, PMID: 34660344 PMC8517479

[ref16] Liu L. Feng Y. Long H. McNally A. Zong Z. (2018). Sequence type 273 carbapenem-resistant *Klebsiella pneumoniae* carrying *bla*_NDM-1_ and *bla*_IMP-4_. Antimicrob. Agents Chemother. 62:e00160. doi: 10.1128/AAC.00160-18, PMID: 29610206 PMC5971603

[ref17] Liu Z. Li J. Wang H. Xia F. Xia Y. Wang H.. (2024). Clonal transmission of *bla*_IMP-4_-carrying ST196 *Klebsiella pneumoniae* isolates mediated by the IncN plasmid in China. J. Glob. Antimicrob. Resist. 38, 116–122. doi: 10.1016/j.jgar.2024.05.002, PMID: 38735531

[ref18] Löhr I. Hülter N. Bernhoff E. Johnsen P. Sundsfjord A. Naseer U. (2015). Persistence of a pKPN3-like CTX-M-15-encoding IncFIIK plasmid in a *Klebsiella pneumonia* ST17 host during two years of intestinal colonization. PLoS One 10:e0116516:e0116516. doi: 10.1371/journal.pone.0116516, PMID: 25738592 PMC4349654

[ref19] Lopez-Diaz M. Ellaby N. Turton J. Woodford N. Tomas M. Ellington M. J. (2022). NDM-1 carbapenemase resistance gene vehicles emergent on distinct plasmid backbones from the IncL/M family. J. Antimicrob. Chemother. 77, 620–624. doi: 10.1093/jac/dkab466, PMID: 34993543

[ref20] Ma Z. Qian C. Yao Z. Tang M. Chen K. Zhao D.. (2024). Coexistence of plasmid-mediated *tmexCD2-toprJ2*, *bla*_IMP-4_, and *bla*_NDM-1_ in *Klebsiella quasipneumoniae*. Microbiol. Spectr. 12:e0387423. doi: 10.1128/spectrum.03874-23, PMID: 39162556 PMC11448383

[ref21] Magobo R. E. Ismail H. Lowe M. Strasheim W. Mogokotleng R. Perovic O.. (2023). Outbreak of NDM-1- and OXA-181-producing *Klebsiella pneumoniae* bloodstream infections in a neonatal unit, South Africa. Emerg. Infect. Dis. 29, 1531–1539. doi: 10.3201/eid2908.230484, PMID: 37486166 PMC10370860

[ref22] Novais A. Ferraz R. V. Viana M. da Costa P. M. Peixe L. (2022). NDM-1 introduction in Portugal through a ST11 KL105 *Klebsiella pneumoniae* widespread in Europe. Antibiotics 11:92. doi: 10.3390/antibiotics11010092, PMID: 35052969 PMC8773016

[ref23] Paczosa M. K. Mecsas J. (2016). *Klebsiella pneumoniae*: going on the offense with a strong defense. Microbiol. Mol. Biol. Rev. 80, 629–661. doi: 10.1128/MMBR.00078-15, PMID: 27307579 PMC4981674

[ref24] Roberts L. W. Catchpoole E. Jennison A. V. Bergh H. Hume A. Heney C.. (2020). Genomic analysis of carbapenemase-producing *Enterobacteriaceae* in Queensland reveals widespread transmission of *bla*_IMP-4_ on an IncHI2 plasmid. Microb. Genom. 6:000321. doi: 10.1099/mgen.0.000321, PMID: 31860437 PMC7067041

[ref25] Sader H. S. Mendes R. E. Carvalhaes C. G. Kimbrough J. H. Castanheira M. (2023). Changing epidemiology of carbapenemases among carbapenem-resistant Enterobacterales from United States hospitals and the activity of aztreonam-avibactam against contemporary Enterobacterales (2019–2021). Open Forum Infect. Dis. 10:ofad046. doi: 10.1093/ofid/ofad046, PMID: 36846612 PMC9945928

[ref26] Sandfort M. Hans J. B. Fischer M. A. Reichert F. Cremanns M. Eisfeld J.. (2022). Increase in NDM-1 and NDM-1/OXA-48-producing *Klebsiella pneumoniae* in Germany associated with the war in Ukraine, 2022. Euro Surveill. 27:2200926. doi: 10.2807/1560-7917.ES.2022.27.50.2200926, PMID: 36695468 PMC9808319

[ref27] Shi Q. Hu H. Yu Q. Huang W. Wang Y. Quan J.. (2024). Chromosomal integration and plasmid fusion occurring in ST20 carbapenem-resistant *Klebsiella pneumoniae* isolates coharboring *bla*_NDM-1_ and *bla*_IMP-4_ induce resistance transmission and fitness variation. Emerg. Microbes Infect. 13:2339942. doi: 10.1080/22221751.2024.2339942, PMID: 38584569 PMC11022923

[ref28] Shu L. B. Lu Q. Sun R. H. Lin L. Q. Sun Q. L. Hu J.. (2019). Prevalence and phenotypic characterization of carbapenem-resistant *Klebsiella pneumoniae* strains recovered from sputum and fecal samples of ICU patients in Zhejiang Province, China. Infect. Drug Resist. 12, 11–18. doi: 10.2147/IDR.S175823, PMID: 30588043 PMC6302810

[ref29] Tacconelli E. Carrara E. Savoldi A. Harbarth S. Mendelson M. Monnet D. L.. (2018). Discovery, research, and development of new antibiotics: the WHO priority list of antibiotic-resistant bacteria and tuberculosis. Lancet Infect. Dis. 18, 318–327. doi: 10.1016/S1473-3099(17)30753-3, PMID: 29276051

[ref30] Wang B. Pan F. Han D. Zhao W. Shi Y. Sun Y.. (2022). Genetic characteristics and microbiological profile of hypermucoviscous multidrug-resistant *Klebsiella variicola* coproducing IMP-4 and NDM-1 carbapenemases. Microbiol. Spectr. 10:e0158121. doi: 10.1128/spectrum.01581-21, PMID: 35019673 PMC8823660

[ref31] Wang Q. Wang X. Wang J. Ouyang P. Jin C. Wang R.. (2018). Phenotypic and genotypic characterization of carbapenem-resistant *Enterobacteriaceae*: data from a longitudinal large-scale CRE study in China (2012–2016). Clin. Infect. Dis. 67, S196–S205. doi: 10.1093/cid/ciy660, PMID: 30423057

[ref32] Wang Q. Wang R. Wang S. Zhang A. Duan Q. Sun S.. (2024). Expansion and transmission dynamics of high risk carbapenem-resistant *Klebsiella pneumoniae* subclones in China: an epidemiological, spatial, genomic analysis. Drug Resist. Updat. 74:101083. doi: 10.1016/j.drup.2024.101083, PMID: 38593500

[ref33] Wen Y. Xie X. Xu P. Yang C. Zhu Z. Zhu J.. (2022). NDM-1 and OXA-48-like carbapenemases (OXA-48, OXA-181 and OXA-252) co-producing *Shewanella xiamenensis* from hospital wastewater, China. Infect. Drug Resist. 15, 6927–6938. doi: 10.2147/IDR.S386345, PMID: 36471715 PMC9719275

[ref34] Woodford N. Turton J. F. Livermore D. M. (2011). Multiresistant Gram-negative bacteria: the role of high-risk clones in the dissemination of antibiotic resistance. FEMS Microbiol. Rev. 35, 736–755. doi: 10.1111/j.1574-6976.2011.00268.x, PMID: 21303394

[ref35] Xiao T. Peng K. Chen Q. Hou X. Huang W. Lv H.. (2022). Coexistence of *tmexCD-toprJ*, *bla*_NDM-1_, and *bla*_IMP-4_ in one plasmid carried by clinical *Klebsiella* spp. Microbiol. Spectr. 10:e0054922. doi: 10.1128/spectrum.00549-22, PMID: 35647621 PMC9241619

[ref36] Zhang Y. Gu D. Yang X. Wu Y. Liu C. Shen Z.. (2021). Emergence and genomic characterization of a KPC-2-, NDM-1-, and IMP-4-producing *Klebsiella michiganensis* isolate. Front. Microbiol. 12:762509. doi: 10.3389/fmicb.2021.762509 35069468 PMC8770907

[ref37] Zhang R. Liu L. Zhou H. Chan E. W. Li J. Fang Y.. (2017). Nationwide surveillance of clinical carbapenem-resistant *Enterobacteriaceae* (CRE) strains in China. EBioMedicine 19, 98–106. doi: 10.1016/j.ebiom.2017.04.032, PMID: 28479289 PMC5440625

[ref38] Zheng Z. Liu L. Ye L. Xu Y. Chen S. (2023). Genomic insight into the distribution and genetic environment of *bla*_IMP-4_ in clinical carbapenem-resistant *Klebsiella pneumoniae* strains in China. Microbiol. Res. 275:127468. doi: 10.1016/j.micres.2023.127468, PMID: 37541025

[ref39] Zhou K. Xiao T. David S. Wang Q. Zhou Y. Guo L.. (2020). Novel subclone of carbapenem-resistant *Klebsiella pneumoniae* sequence type 11 with enhanced virulence and transmissibility, China. Emerg. Infect. Dis. 26, 289–297. doi: 10.3201/eid2602.190594, PMID: 31961299 PMC6986851

[ref40] Zhu Y. Liu W. Schwarz S. Wang C. Yang Q. Luan T.. (2020). Characterization of a *bla*_NDM-1_-carrying IncHI5 plasmid from *Enterobacter cloacae* complex of food-producing animal origin. J. Antimicrob. Chemother. 75, 1140–1145. doi: 10.1093/jac/dkaa010, PMID: 32016414

[ref41] Zhu Z. Xie X. Yu H. Jia W. Shan B. Huang B.. (2023). Epidemiological characteristics and molecular features of carbapenem-resistant *Enterobacter* strains in China: a multicenter genomic study. Emerg. Microbes Infect. 12:2148562. doi: 10.1080/22221751.2022.2148562, PMID: 36382635 PMC9769138

